# The role of radiolabeling in BNCT tracers for enhanced dosimetry and treatment planning

**DOI:** 10.7150/thno.88998

**Published:** 2023-09-25

**Authors:** Sajid Mushtaq, Park Ji Ae, Jung Young Kim, Kyo Chul Lee, Kwang Il Kim

**Affiliations:** 1 Division of Applied RI, Korea Institute of Radiological & Medical Sciences (KIRAMS) Seoul 01812, Republic of Korea.; 2 Department of Nuclear Engineering, Pakistan Institute of Engineering and Applied Sciences, P. O. Nilore, Islamabad 45650, Pakistan.

**Keywords:** BNCT, PET, SPECT, radiolabeling, theranostics, targeted therapy

## Abstract

Positron emission tomography (PET) and single photon emission computed tomography (SPECT) are potent technologies for non-invasive imaging of pharmacological and biochemical processes in both preclinical and advanced clinical research settings. In the field of radiation therapy, boron neutron capture therapy (BNCT) stands out because it harnesses biological mechanisms to precisely target tumor cells while preserving the neighboring healthy tissues. To achieve the most favorable therapeutic outcomes, the delivery of boron-enriched tracers to tumors must be selective and efficient, with a substantial concentration of boron atoms meticulously arranged in and around the tumor cells. Although several BNCT tracers have been developed to facilitate the targeted and efficient delivery of boron to tumors, only a few have been labeled with PET or SPECT radionuclides. Such radiolabeling enables comprehensive *in vivo* examination, encompassing crucial aspects such as pharmacodynamics, pharmacokinetics, tumor selectivity, and accumulation and retention of the tracer within the tumor. This review provides a comprehensive summary of the essential aspects of BNCT tracers, focusing on their radiolabeling with PET or SPECT radioisotopes. This leads to more effective and targeted treatment approaches which ultimately enhance the quality of patient care with respect to cancer treatment.

## 1. Introduction

**Figure 1:**
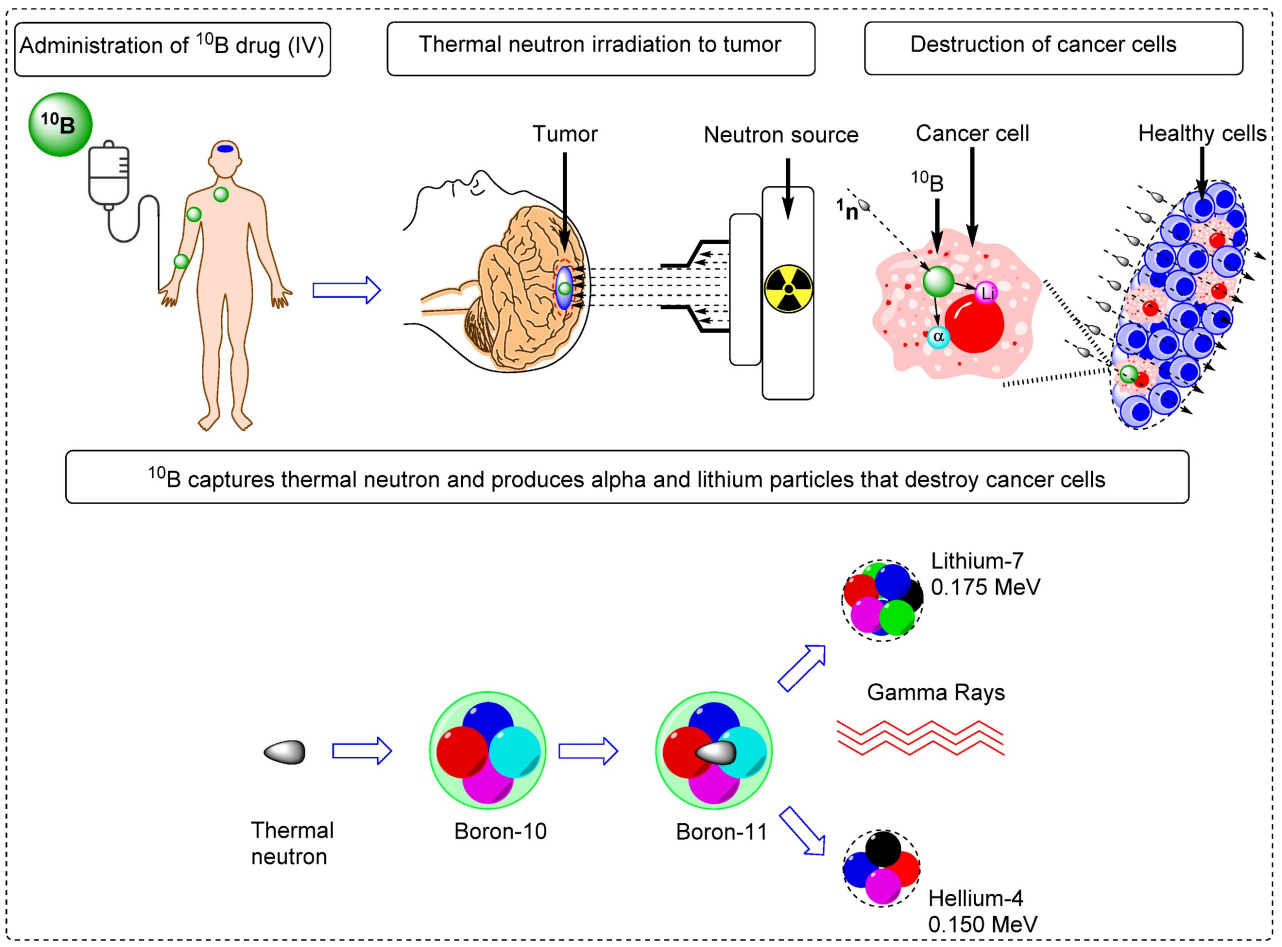
Schematic overview of the BNCT principle and its impact on tumor cell improvement.

Boron neutron capture therapy (BNCT) represents a remarkable advancement in radiation therapy. It utilizes biological mechanisms to selectively target tumor cells while sparing the neighboring healthy tissues. It is a highly efficient targeted radiotherapy technique with higher therapeutic benefits than those of traditional radiotherapy 1, 2. Various micro- and macro biologically active molecules have been utilized as tracers for BNCT. These tracers are enriched with Boron-10 (^10^B) isotopes because of their high interaction cross-sections of 3,837 barns with thermal neutrons 3. Thermal neutron and ^10^B interactions yield unstable ^11^B isotopes that undergo nuclear reactions to produce highly active ^7^Li (175 keV/μm) and ^4^He particles (150 keV/μm), along with gamma rays (Figure 1) 4. In a biological system, these particles with high linear energy transfer (LET), ^7^Li (175 keV/μm), and α particles (150 keV/μm), deposit their energy along their path (5-8 μm), which is shorter than the average cell diameter 5, 6. It is possible to treat subclinical lesions using tumor-specific boron-containing compounds because BNCT is a biologically, not a geometrically, targeted modality 7. Nevertheless, the precise timing of thermal neutron irradiation plays a pivotal role in BNCT, as it determines the optimal concentration of ^10^B within the tumor relative to healthy tissues, thereby achieving the maximum tumor-to-normal tissue (T/N) ratio 8.To accomplish this, it is imperative to employ boron tracers that exhibit an appropriate retention time in the tumor and demonstrate an accumulation of boron that is at least three times higher in the tumor than in the background tissues. Moreover, maintaining a tumor boron concentration of ≥20 ppm is crucial for successful BNCT outcomes 9.

**Figure 2:**
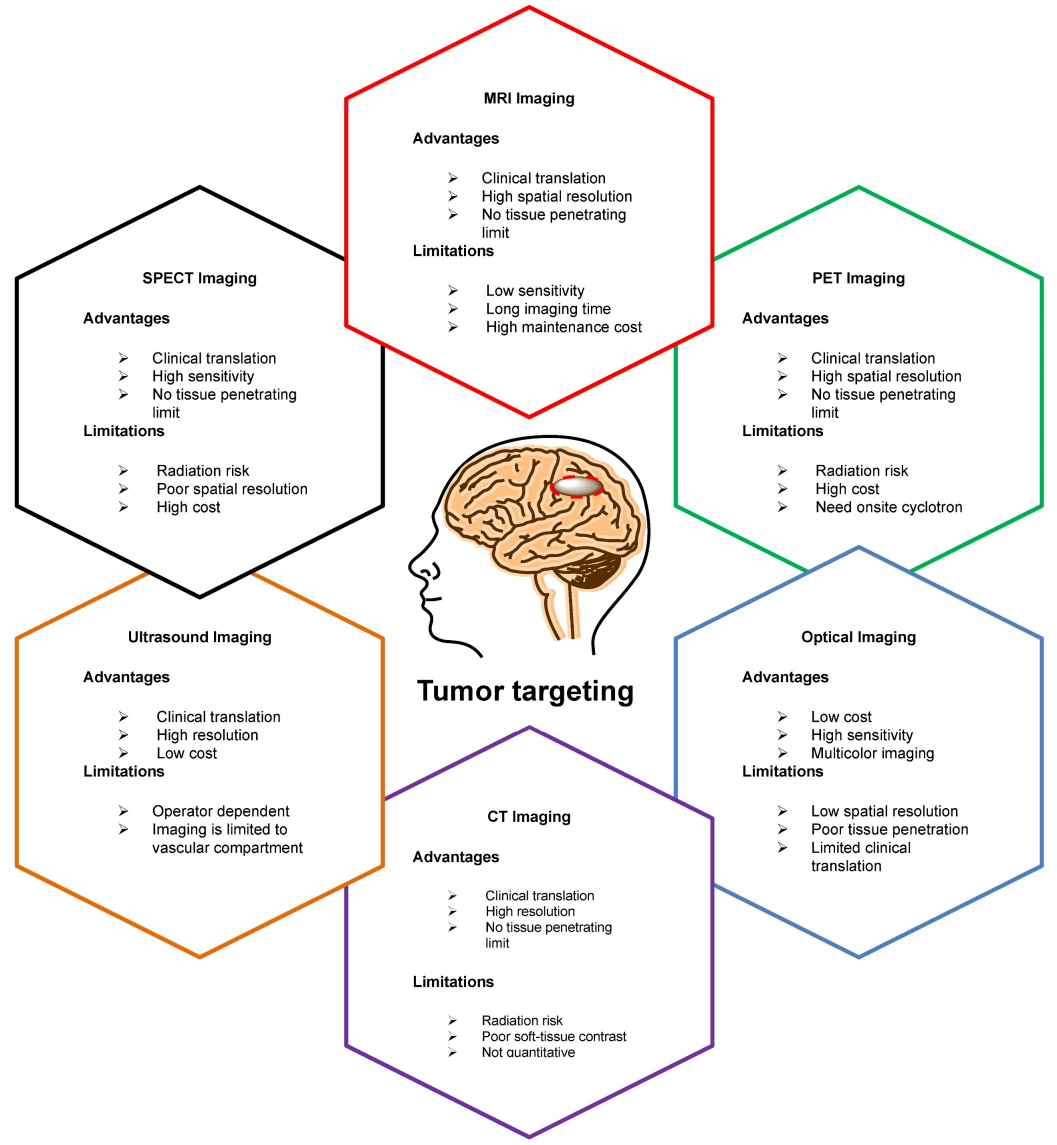
A schematic overview of various imaging modalities, highlighting their unique capabilities, strengths, and limitations. PET and SPECT exhibit several promising advantages over traditional imaging technologies.

To advance the development of BNCT as a viable clinical modality, several tumor-targeting drugs (Table 1) have been used for the efficient delivery of ^10^B isotopes to the tumors 10-30. Irradiation of ^10^B-containing tumors with high-intensity thermal neutron beams generates an intricate distribution of doses in the tissues. However, the absorbed dose pattern cannot be explained using conventional concepts 31. This is attributed to the inhomogeneous release of energy from the targeted tissue. Considering the small range of ^7^Li and ^4^He particles, the effectiveness of BNCT depends on the precise location and accumulation of boron within the tumor cells 32. Therefore, it is imperative to study the physiology, pharmacokinetics, organ distribution, retention, and excretion of ^10^B in the patients 33. To achieve this, appropriate techniques and technologies that are capable of accurately quantifying the amount of boron in various bodily compartments and body fluids, such as urine and blood, are required. Additionally, imaging technologies that are capable of visualizing the distribution of ^10^B at the cellular level should be employed to provide valuable information regarding its localization within tumor cells and surrounding tissues 34. Non-invasive techniques are considered optimal for determining^10^B concentrations *in vivo*; therefore, molecular imaging has emerged as an effective solution 35. Imaging modalities such as single photon emission computed tomography (SPECT) and positron emission tomography (PET), offer distinct advantages over conventional techniques (Figure 2) by providing high-resolution information without compromising the integrity of the organism 36-40. Importantly, PET and SPECT can achieve sufficient spatial resolution, ensuring the accurate characterization of the distribution and concentration of ^10^B in intact organism. Several general reviews have been published to discuss the progress in the field of BNCT 41-49. However, this review provides a comprehensive overview of the latest and the most efficient methodologies for synthesizing and radiolabeling BNCT probes for therapy, as well as *in vivo* imaging using PET- or SPECT-based radioisotopes. Furthermore, it provides detailed pharmacokinetic results at the preclinical or clinical stages.

## 2. Direct and indirect radiolabeling of BNCT tracers

**Figure 3:**
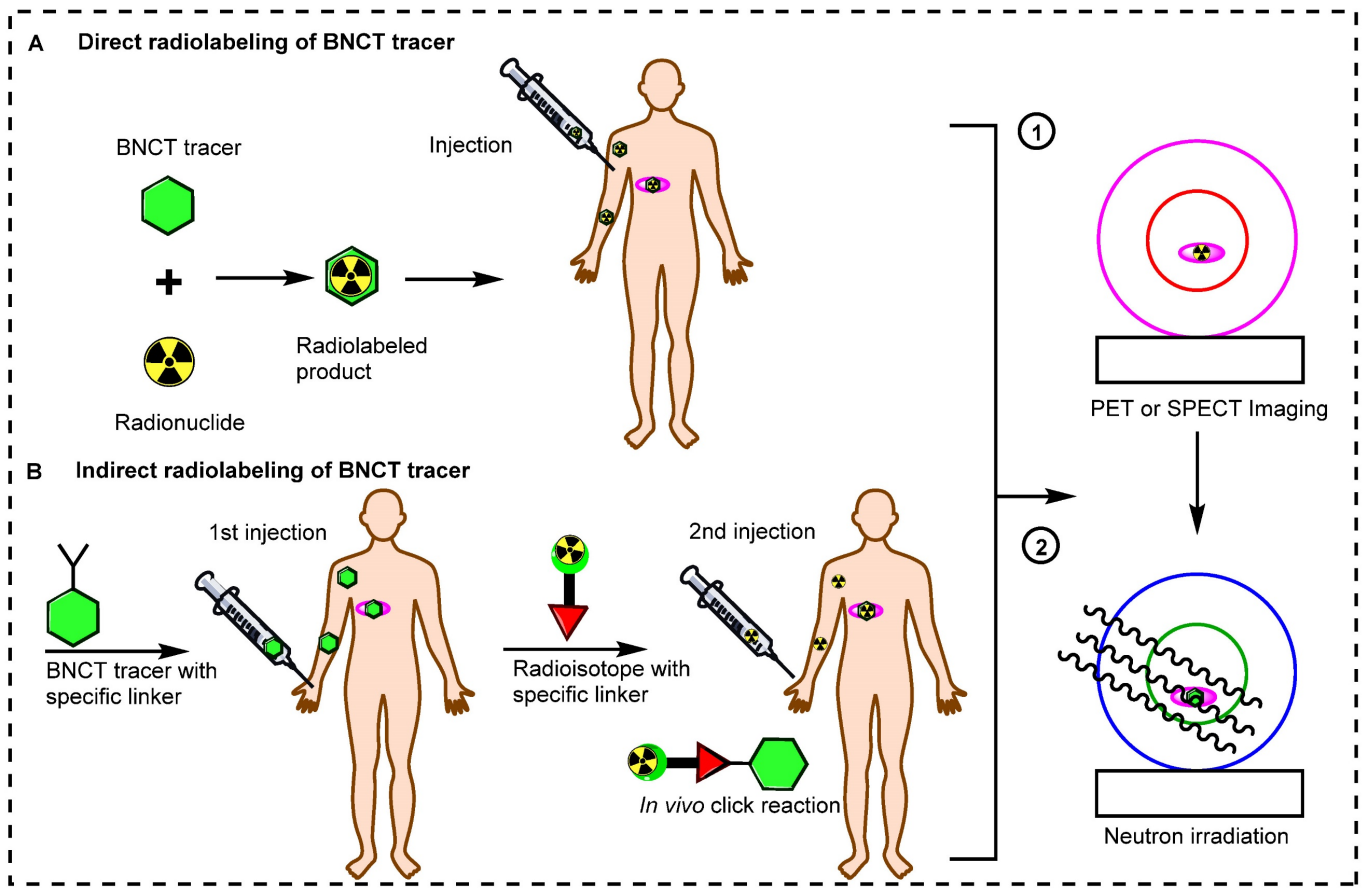
Schematic illustration of radiolabeling strategies for molecular imaging. (A) Direct radiolabeling of BNCT tracers. (B) Indirect radiolabeling of BNCT tracers.

Small BNCT tracers have been synthesized and directly radiolabeled for noninvasive molecular imaging. Initially, the (L)-4-dihydroxy-borophenylalanine (BPA) and sodium mercaptoundecahydro-closo-dodecaborate (BSH) BNCT tracers were directly radiolabeled and studied in preclinical and clinical settings 50,51. These small molecules have fast pharmacokinetics, and after the targeted delivery of boron to the tumor, these tracers are immediately excreted from the body. Recently, antibodies and inorganic and organic nanoparticles have been used to synthesize BNCT tracers 52. These tracers have gained widespread acceptance for boron delivery because of their specificity and high affinity for tumors. However, the use of these BNCT precursors is limited by their comparatively large size, which results in slow accumulation in tumors and slow excretion from the body. Owing to their slow pharmacokinetics, a long time is needed to achieve an adequate boron accumulation in the tumor and a high T/N ratio. Long-lived radionuclides have been used to address these issues 53. However, these radionuclides can emit high radiation doses to the healthy tissues, especially the bone marrow. In recent decades, several novel methodologies have emerged to address the pharmacokinetic limitations of large BNCT tracers. An alternative approach is the indirect radiolabeling of the tracer *in vivo* (Figure 3) 54, 55. In this pre-targeting strategy, the targeting tracer is injected into the body. Once it reaches maximum accumulation in the tumor and is mostly cleared from healthy tissues, a complimentary small molecule radiolabeled with a suitable radioisotope is administered for the *in vivo* click reaction 56, 57. The indirect radiolabeling strategy allows for the efficient quantification of the BNCT tracer within the tumor with significantly low radiation doses to healthy tissues.

## 3. Radionuclides used for PET and SPECT imaging of BNCT tracers

In nuclear imaging, PET and SPECT are extensively used to determine the pharmacokinetics and dosimetry of radiolabeled tracers of medical interest 58. These imaging technologies are capable of visualizing the *in vivo* behavior. The quantitative monitoring of radiolabeled BNCT tracers for several hours, days, or even weeks provided a suitably long biological and physical half-life of the radionuclide (Table 2) 59-60. SPECT technology is based on the quantification of gamma-ray photons emitted by radioisotopes such as radioiodine (^123^I or ^125^I), indium (^111^In) or technetium (^99m^Tc) 61. SPECT detectors record the absorbed photons, in two-dimensional projections. By processing these 2D images captured from various angles, it is possible to reconstruct the required 3D images 62. PET imaging is performed by detecting positrons emitted by beta-emitting radioisotopes, such as fluorine (^18^F), copper (^64^Cu), gallium (^68^Ga), and zirconium (^89^Zr) 63. The PET imaging system based on the detection of a pair of 511 keV gamma-ray photons produced by positron electron annihilation. Scintillator detectors convert gamma rays into visible light photons, which are detected by photomultiplier tubes. Afterwards, the data are processed and converted into 3D images 64. PET imaging has demonstrated better sensitivity and spatial resolution (2-4mm) than those of SPECT imaging (4-6mm) in clinical settings. Moreover, PET imaging enables the quantitative determination of radiotracer uptake in the tissue of interest, which is a challenge for SPECT owing to its low spatial resolution 65. PET and SPECT imaging techniques can be integrated with other imaging modalities, such as computed tomography (CT) and magnetic resonance imaging (MRI), to acquire more comprehensive anatomical information 66. Moreover, the *in vivo* stability of radiolabeled compounds plays a pivotal role in molecular imaging because, in their free state, radioisotopes can be distributed in tumors and other healthy tissues leading to spurious signals (Table 3).

## 4. Radionuclides used for PET imaging of BNCT tracers

### 4.1 ^18^F labeled BNCT tracers

**Figure 4:**
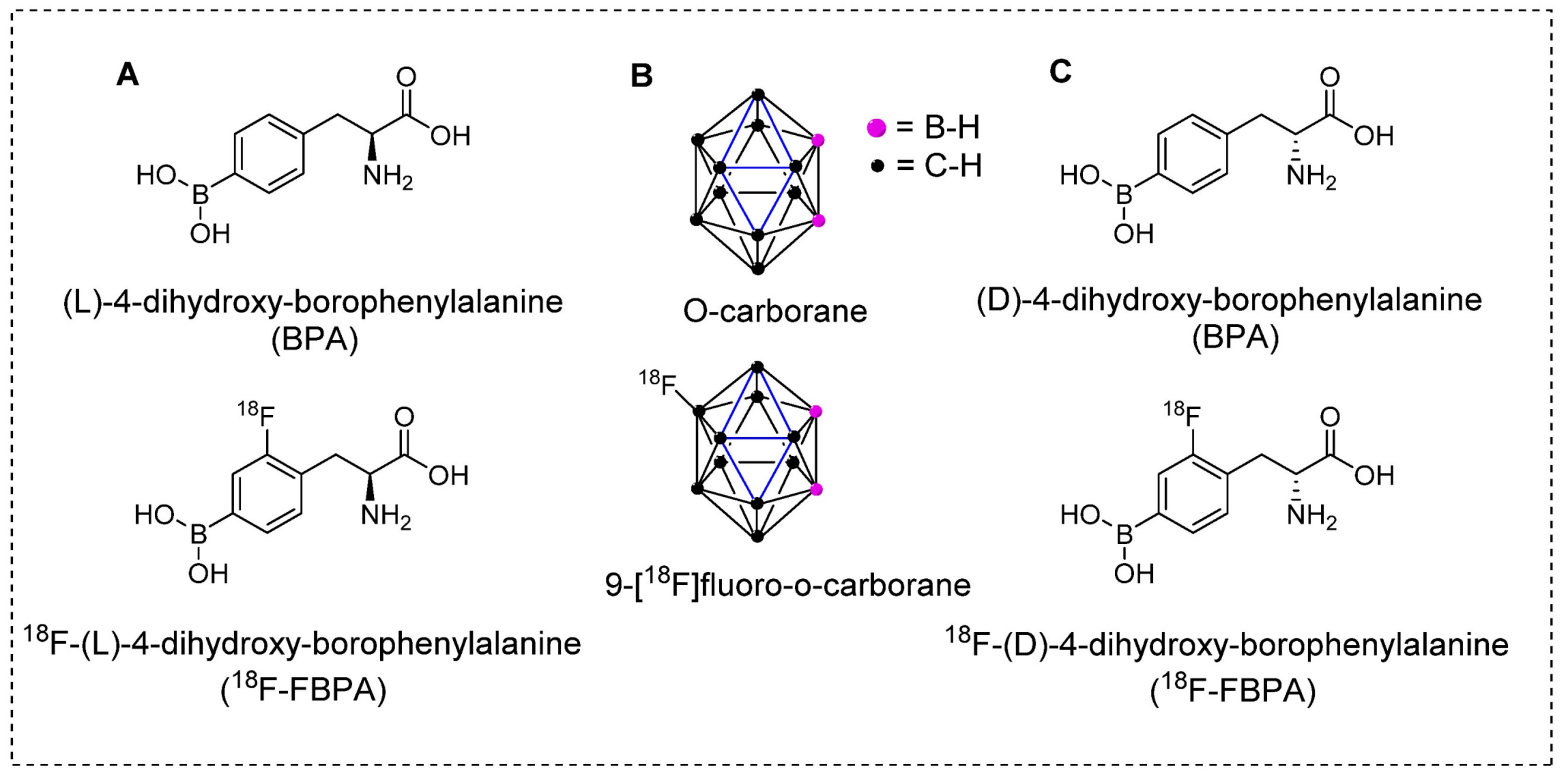
Introducing the pioneering and highly effective BNCT tracers. (A) Structure of L-BPA and corresponding PET tracers. (B) Structure of O-carborane and corresponding PET tracer. (C) Structure of D-BPA and corresponding PET tracer.

The crucial step towards producing therapeutically beneficial BNCT is the effective and targeted delivery of ^10^B atoms to cancerous cells at higher concentrations than those delivered to background tissues 67. Several tumor-specific carriers have been developed and tested to achieve this goal. In the first era of BNCT, the BPA and BSH tracers (Figure 4) showed promising results. For instant, BPA demonstrated high tumor accumulation, low toxicity, and higher tumor retention times than those of the other tracers under testing. Although these BNCT tracers show nonspecific uptake by tumors, the^10^B installed phenylalanine derivative has been used in clinical trials in the United States and Japan 68.

**Scheme 1:**
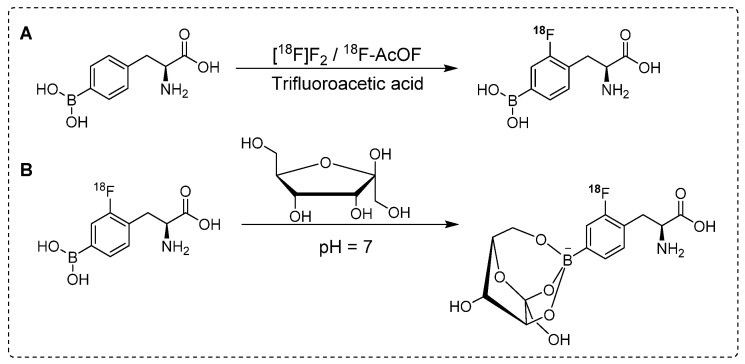
Synthesis of^18^F labeled ^18^F-FBPA analogues. (A) Electrophilic substitution reaction for the radiofluorination of (L)-4-dihydroxy-borophenylalanine using ^18^[F]-F_2_ or ^18^F-acetylhypofluorite (^18^F-AcOF). (B) Synthesis of ^18^F-FBPA-fructose complex utilizing fructose at neutral pH: Enhancing water solubility for superior biocompatibility.

For complete *in vivo* imaging and detailed pharmacokinetic studies, Ishiwata and co-workers synthesized ^18^F-FBPA (Scheme 1A). To our knowledge, that was the first *in vivo* PET imaging study using radiolabeled BPA. In that study, a structural analog of boronophenylalanine (L-BPA) was radiolabeled via the electrophilic substitution of radio-fluorine on borono-acid-containing aromatic ring.^18^F-FBPA was used to study the boron concentration in brain tumors. The radiolabeled compound showed enhanced lipophilicity compared to that of its predecessor 69. It accumulated in the liver, spleen, pancreas, intestine and testes 10 min after the intravenous injection; however, the radioactivity signal decreased over time. The brain uptake showed an increase with time and was observed to reach maximum level at 2 hr postinjection and remained consistent afterwards. One hr postinjection, a considerable amount of tracer was detected in the urine. PET imaging showed promising features of the radiotracer and the tumor accumulation and retention were high. Owing to the rapid washout from the background tissues, high tumor-to-background (T/B) ratios were observed 1 hr postinjection. It was concluded that for maximum therapeutic benefits, thermal neutron irradiation should be applied 1.5 hr postinjection 70. BPA has several limitations when used as a BNCT tracer; one of the prominent drawbacks is its poor water solubility. To address this limitation, complexation of BPA with fructose is considered a potential solution. Kabalka and co-workers and later Imahori and co-workers synthesized ^18^F-FBPA using electrophilic substitution reaction and fructose was used for complexation of BPA to enhance the solubility 71, 72. The ^18^F-FBPA complex with fructose (Scheme 1B) exhibited improved solubility; nevertheless, its biodistribution profile was differed from that of ^18^F-FBPA and BPA when administered alone 72. Clinical trials have been performed in patients with glioblastoma. The PET-based quantification studies revealed that BPA complex with fructose showed more complex pharmacokinetics than that of its peer amino acids, and it was similar to that of ^18^F-fluorodeoxyglucose (^18^F-FDG). The procedure provided ^18^F-FBPA compound but, with low specific activity. This could be possible because radio-fluoride adhered to the targeting material, and carrier-added fluorine gas was used to recover the radio-fluoride. The specific activity was 1,000 times less than that of nucleophilic substitution reaction. Several conditions were investigated to improve the radiochemical yield and specific activity. The BPA and fluorine gas concentrations were optimized, various eluent were used for better HPLC separation, and the concentration of fluorine as the carrier gas in the target was optimized. However, no significant improvements were observed 72.

**Scheme 2:**
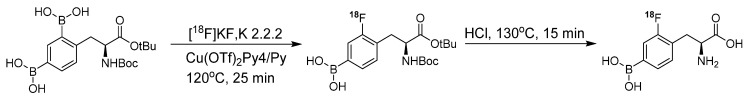
Synthesis of ^18^F-Labeled BPA analogue (^18^F-FBPA) through nucleophilic radio-fluorination reaction with diborono precursor for enhanced molecular imaging capabilities.

The conventional nucleophilic radio˗fluorination reaction is highly viable; however, the undesirable fluorodeboronation hampers this approach. To exploit the advantages of this approach, diboronos were installed on an aromatic ring.^18^F-FBPA was obtained by replacing the boron group at the ortho position. This selective radio˗fluorination used one boron group to install ^18^F for PET imaging, whereas the other boron group remained intact for therapeutic studies (Scheme 2). The radiolabeling reaction provided ^18^F-FBPA with high specific activity and minimal structural changes in the parent BPA structure; however, the accumulation of ^18^F-FBPA in the organs varied depending on the mode of injection. This phenomenon could be explained by the fact that achieving high˗contrast images requires only a minute amount of ^18^F˗FBPA. However, for BNCT, a high tracer concentration, *i.e.* as several milligrams per gram of body weight, is necessary. Unlike the bolus injection method used for ^18^F-FBPA, BNCT requires continuous intravenous administration of BPA over an extended time period. However, clinical studies have demonstrated that comparable results can be obtained using BNCT dosimetry.^18^F-FBPA exists as a pair of enantiomers, namely the D-and L-isomers. The isomers exhibit distinct pharmacokinetics in humans. In preclinical studies, D-isomers demonstrated superior tumor uptake with a higher T/B ratio (6.93) compared to that of L-isomer, which yielded a ratio of 1.45 in a glioma model 73. This was attributed to the enhanced washout of the D-isomer from the background tissues, resulting in a higher contrast ratio. Another notable characteristic of the D-isomer is its excretion through the kidneys, which facilitates visualization of the abdominal area. Further *in vivo* studies on the D-isomer of ^18^F-FBPA are warranted to explore its potential for improved therapeutic benefits through BNCT treatment 73.

**Figure 5:**
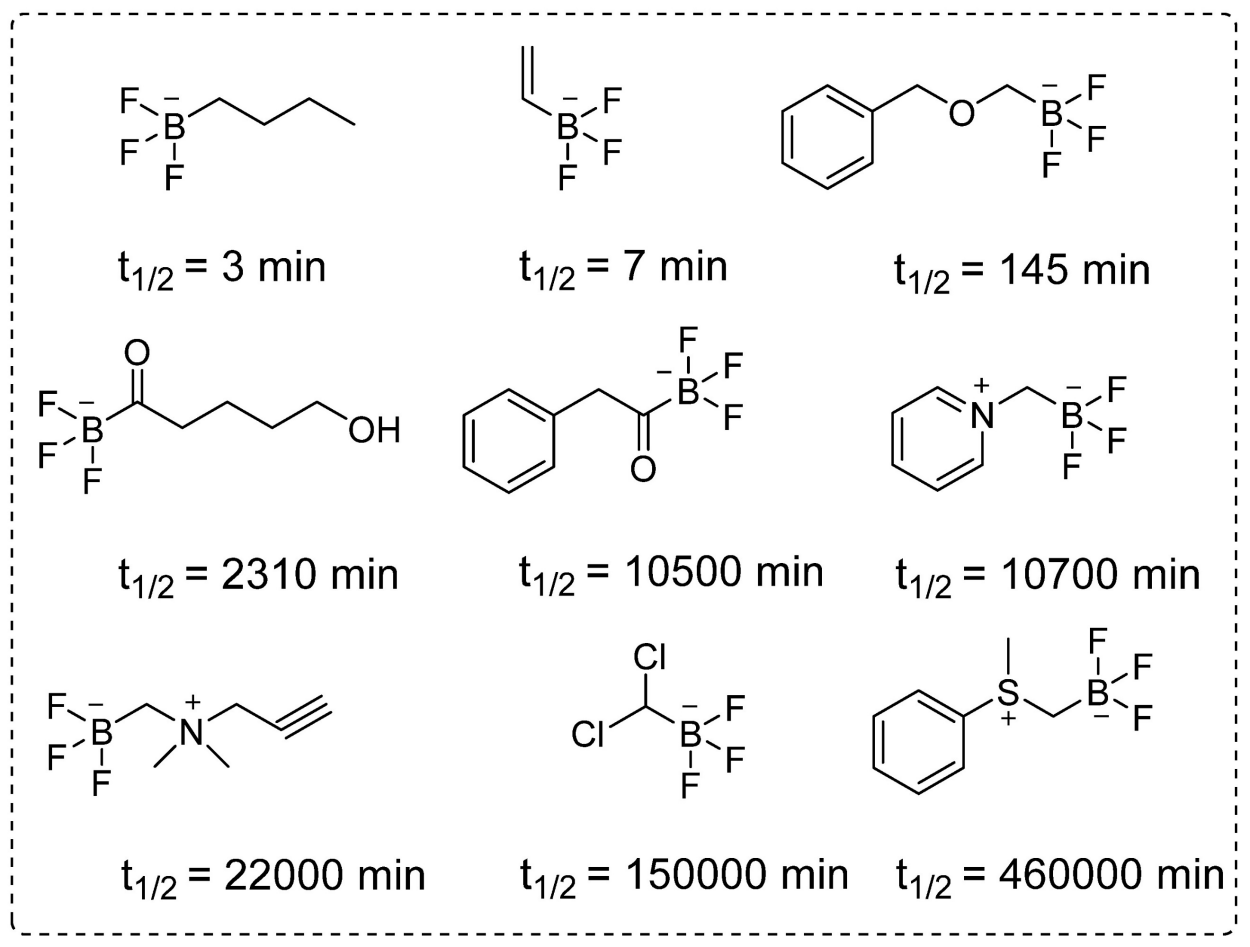
The structural diversity and half-lives of alkyl- and acyltrifluoroborates: Unlocking the promising potential for BNCT applications.

**Figure 6:**
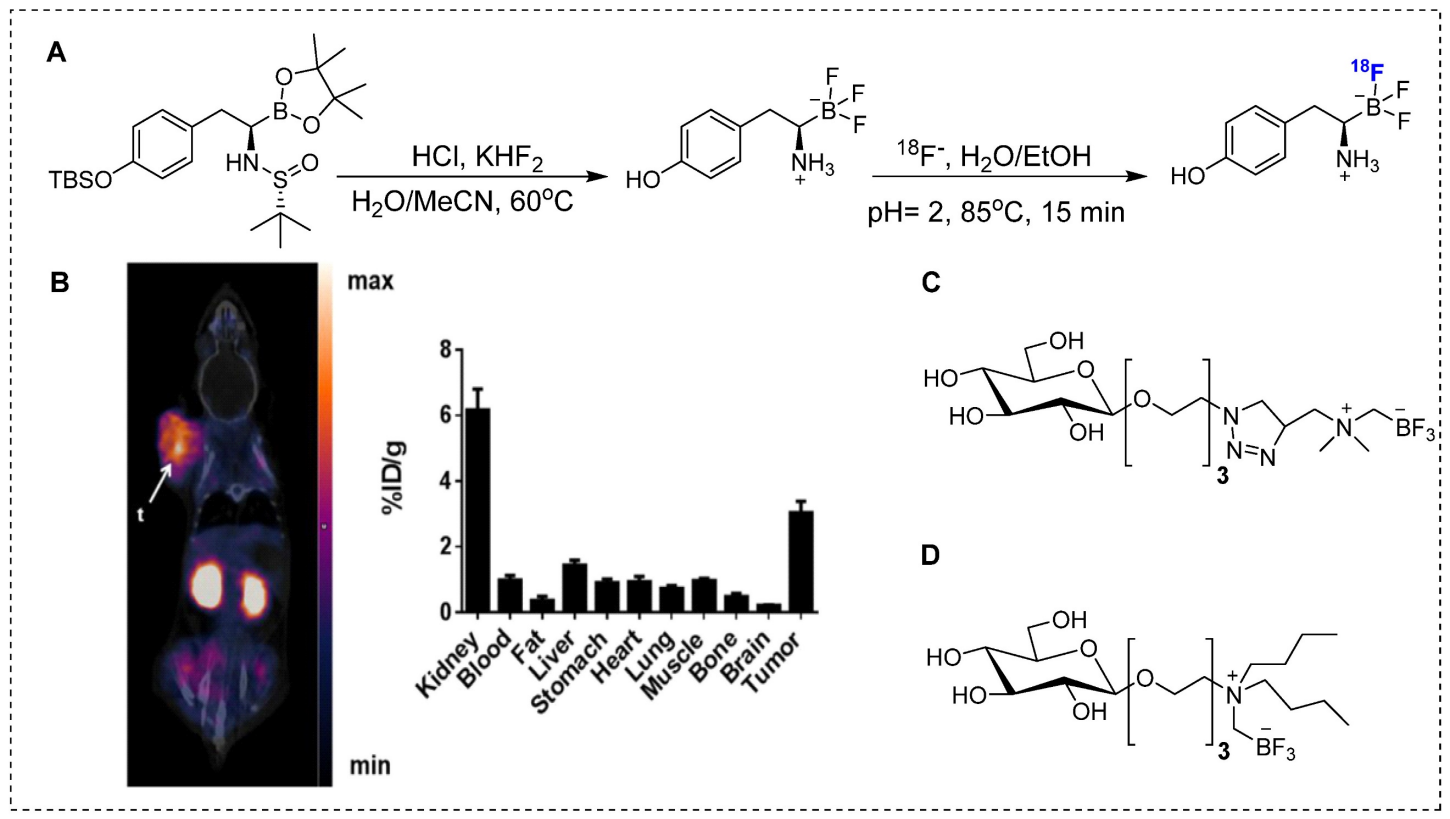
Synthesis and preclinical evaluation of organotrifluoroborates (A) Detailed radiosynthesis of ^18^F labeled fluoroboronotyrosine ^18^F-FBY.(B)B16-F10 tumor bearing mouse was injected with ^18^F-FBY, and a PET image was acquired 1 hr postinjection. ^18^F-FBY exhibited high tumor accumulation compared to that of the healthy tissues. The biodistribution study (n = 4), which was conducted 1 hr postinjection utilizing B16-F10 tumor-bearing mice, further supports the PET imaging data. Adapted with permission from 75, copyright 2019, American Chemical Society. (C, D) Representative organotrifluoroborates sugar conjugate.

Despite several promising results, inadequate tumor targeting and metabolic instability have persisted since the initial application of ^18^F-FBPA. To ensure better therapeutic results, trifluoroborate functional group-based tracers for BNCT were synthesized. The use of trifluoroborate-based structures provided stable boron for targeting in BNCT, while simultaneously enabling PET imaging through ^18^F labeling via ^18^F to ^19^F exchange reaction. For example, a boronic ester can be transformed into its corresponding trifluoroborate using potassium hydrogen fluoride under acidic conditions. The ^18^F radiolabeling was achieved through radio-fluoro exchange reaction, resulting in a high radiochemical yield (>50%) in just 15 min. Several aliphatic and aromatic tracers containing trifluoroborate groups have been synthesized using this strategy. The initial screening revealed that the stability of aromatic trifluoroborates depends on various factors, including the presence of electron-withdrawing groups at the ortho and para positions (Figure 5). For instance, the substitution of a trialkylphosphonium salt significantly prolongs the stability of trifluoroborate, maintaining it for more than 3,397 hr 74. Similarly, zwitterionic amino substitution has been introduced to synthesize stable aliphatic trifluoroborates. The reported fluoroboronotyrosine (FBY) was radiolabeled with^18^F via an exchange reaction (Figure 6A). After HPLC purification, a radiochemical yield of 30% was achieved for radiolabeled FBY, while maintaining radiochemical purity of over 99%. This novel BNCT tracer exhibited excellent stability under oxidative conditions. After continuous interaction with an H_2_O_2_ solution for 4 hr, the compound exhibited 98% stability. In contrast to ^18^F-FBY, ^18^F-FBPA showed 99% conversion to a byproduct within 1 hr 75. Interestingly, a boron concentration of 19.59 ppm was observed in a mouse xenograft model using ^18^F-FBY. The high T/N ratio confirmed the potential of ^18^F-FBY as a PET imaging agent (Figure 6B). However, a high concentration of boron is required in tumors to meet the therapeutic agent criteria. Compared with BPA, FBY offers several advantages, including high stability and water solubility, without the need for auxiliary groups such as fructose. During clinical trials, ^18^F-FBY exhibited high uptake in both low and high-grade tumors. However, the second important factor, which is the concentration of the tracer or the boron level, has not yet been determined in humans 76. In another example, Confalonieri *et al*. synthesized a library of BNCT tracers using various combinations of monosaccharide and ammonium trifluoroborate groups. Among the available structures, triazole-based conjugates (Figure 6C) met the cytotoxicity criteria. Compound Glc1 (Figure 6D) was selected for ^18^F radiolabeling and *in vivo* study. However, the radiolabeled precursor showed poor stability and low brain uptake as compared with ^18^F-FDG and could not be further evaluated for BNCT 77.

The electrophilic fluorination technique was also investigated using non-glioma BNCT tracers, including boronofenbufen, which belongs to the class of non-steroidal anti-inflammatory drugs. Radio˗fluorinated analog of boronofenbufen have been used for imaging cholangiocarcinoma, a type of cancer that is often challenging to diagnose due to its occurrence inside the liver. Electrophilic fluorination of boronofenbufen yielded two enantiomers in moderate radiochemical yields. However, PET images confirmed a low T/B ratio, indicating that it is not a suitable imaging agent for cholangiocarcinoma imaging or BNCT therapy 78. Although multiple^18^F˗labeled BNCT compounds have been developed, the short half˗life of ^18^F (110 min) limits its suitability as a radioisotope. Therefore, the use of long˗lived radioisotopes becomes imperative to comprehensively assess the detailed biodistribution and tumor uptake of BNCT tracer.

### 4.2 ^64^Cu labeled BNCT tracers

**Figure 7:**
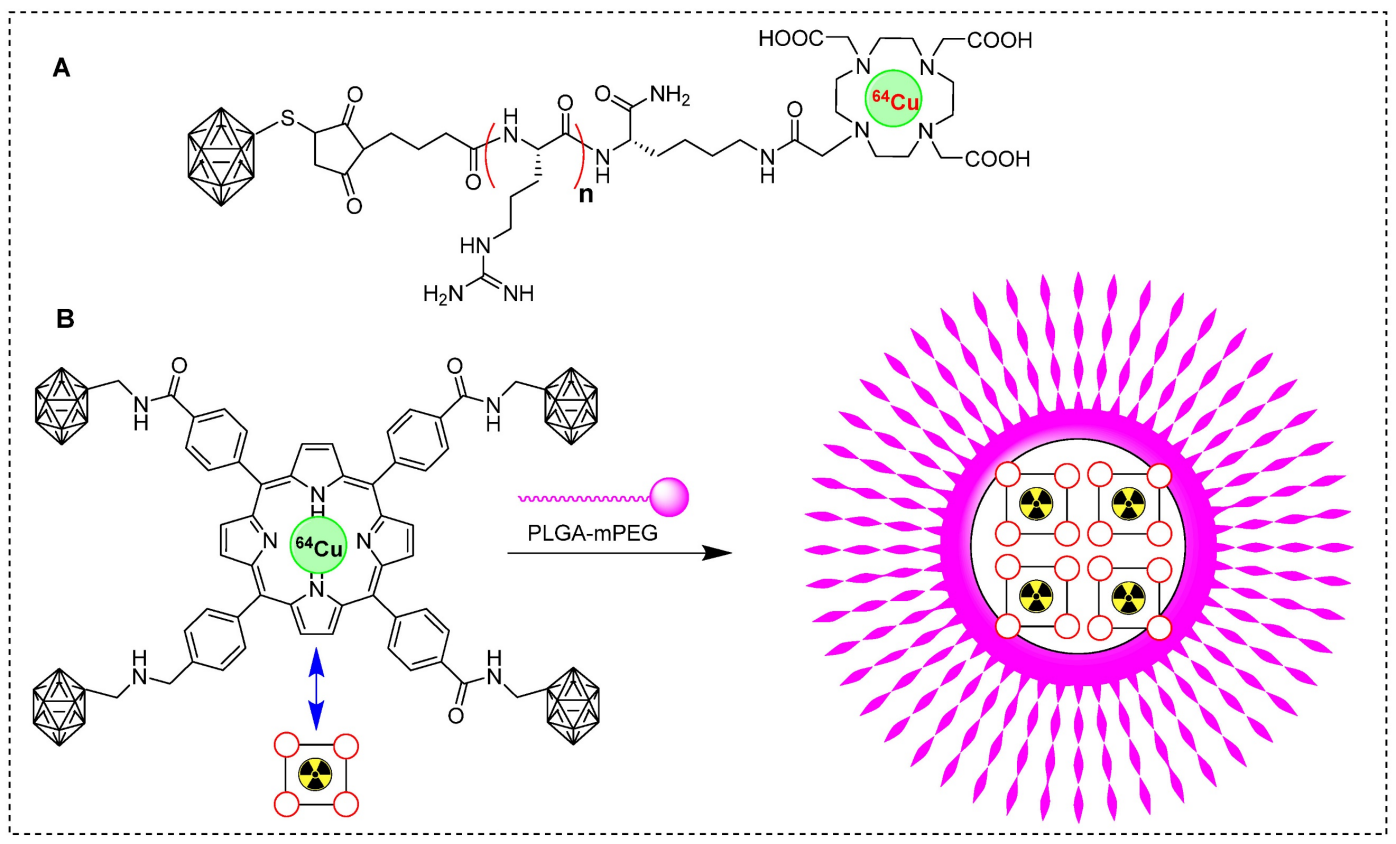
Schematic representation of ^64^Cu labeled BNCT tracers. (A) ^64^Cu labeled BSH-n(R)-DOTA precursor (n= 0, 2, 3; R= Arg). (B) Schematic illustration of synthesis of ^64^Cu-labeled boronated porphyrins nanoparticles.

Copper-64 is one of the most promising clinically used PET radioisotopes 79. Several studies reported radiolabeling of BNCT tracers using ^64^Cu to study their pharmacokinetics. In one study, glioblastoma was treated with BNCT and BSH as a boron source. The *in vivo* pharmacokinetics of ^64^Cu˗labeled BSH and its analog were monitored. BSH-DOTA exhibited intercellular accumulation, but demonstrated insufficient therapeutic benefit. Interestingly, the addition of short arginine residues BSH-nR-DOTA (Figure 7A) increased the cellular uptake of boron, as indicated by *in vivo* and *in vitro* studies. The *in vivo*, boron distribution was studied 24 hr postinjection and PET images showed BSH-3R-DOTA localization in both the tumor and its surrounding periphery. However, BSH-DOTA and BSH-2R-DOTA showed minimal accumulation in the tumor area. Interestingly, the tumor uptake of ^64^Cu-BSH-3R-DOTA was 2.25, 2.38, and 2.97 %ID/cc at 6, 12, and 24 h, respectively. This uptake was highly selective for the tumor, indicating the targeted nature of the compound. The T/N brain ratios at 6 and 24 h postinjection were 15.5 and 8.2, respectively, for ^64^Cu-labeled BSH-3R-DOTA, and 3.1 and 3.7, respectively, for ^64^Cu-labeled BSH-DOTA. The strategy of labeling with ^64^Cu was efficient in identifying a suitable BSH tracer for BNCT 80. Recently, some groups have explored the potential of boronated porphyrins to identify the best boron delivery tracers. Porphyrins have demonstrated various preclinical advantages, including high specificity in targeting tumor cells, prolonged retention, and potential for photodynamic therapy and BNCT 81, in the *in vivo* imaging using fluorescence, PET or SPECT imaging 82-83. Several boronated porphyrins have exhibited promising results in preclinical studies. However, direct toxicity of platelets has hampered their use in clinical studies 84. In a study, boronated porphyrins have been modified with PLGA-mPEG micelles to mitigate toxicity, resulting in a new formulation called boronated porphyrin nanoparticles (BPNs). BPNs were radiolabeled with ^64^Cu to investigate tumor accumulation and pharmacokinetic before neutron irradiation (Figure 7B) 85.^64^Cu-BPN exhibited significant tumor accumulation in xenografted mice and PET imaging facilitated excellent visualization for monitoring the boron concentration in the tumor. PET imaging confirmed the advantages of administrating multiple bolus injections at lower tracer concentrations over a single injection at a high concentration. Multiple injections resulted in a higher concentration of boron in the tumor (>100 ppm), and T/B ratio was exceptionally high (>50%). BNCT, with the assistance of ^64^Cu-BPN tracer, completely cured B16-F10 tumors in the animals. However, intravenous injection of^64^Cu-BPNswas associated with plasma instability and an early release of boronated porphyrins.

**Figure 8:**
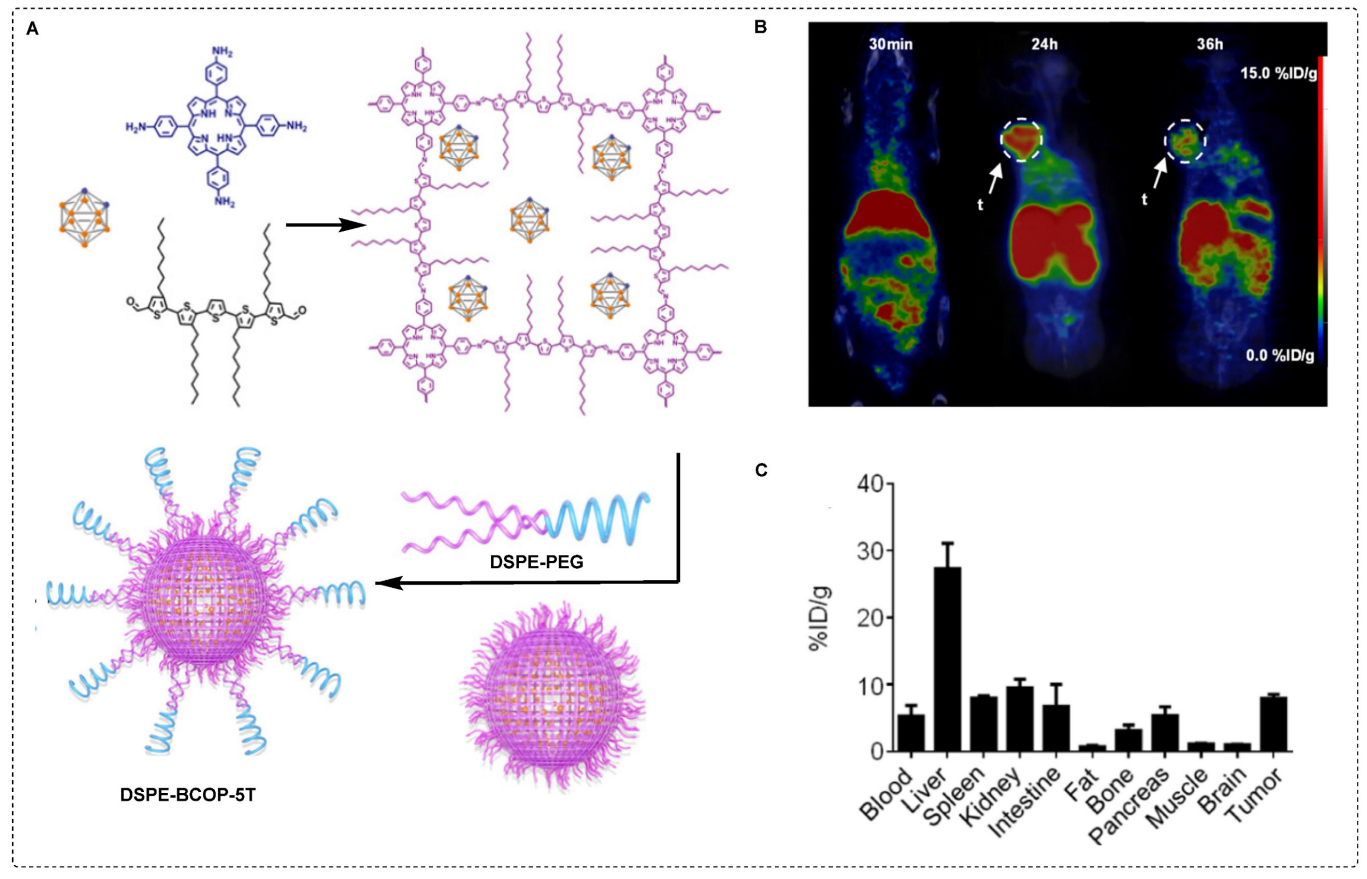
^64^Cu labeled DSPE-BCOP-5T and preclinical evaluation. (A) Schematic illustration of the synthesis of DSPE-BCOP-5T BNCT tracer. (B) PET/CT images have been obtained for ^64^Cu-DSPE-BCOP-5T at 30 min, 24, and 36 hr postinjection using 4T1 tumor-bearing mice. High tumor uptake is indicated by white arrows (n = 3). (C) Biodistribution data was acquired for ^64^Cu-DSPE-COP-5T in 4T1 tumor-bearing mice 24 hr postinjection (mean ± SE, %ID/g) (n = 6). Adapted with permission from 86, copyright 2020, American Chemical Society.

To achieve better stability, carborane was immobilized on covalent organic polymers (COPs) and a porphyrin structure was used for the chelation of ^64^Cu. The complex was further modified with DSPE-PEG to enhance its stability and solubility in the aqueous phase (Figure 8A). The tracer ^64^Cu labeled DSPE-BCOP-5T was evaluated in animal models. It was considered to be sufficient despite exhibiting poor stability in fetal bovine serum compared to phosphate-buffered saline. Some *in vivo* assays revealed a significant tumor uptake after 24 hr (55.24 ± 2.13 ppm) accompanied by intense signals from other organs. Following a single bolus injection, the ratios of T/N and T/B were determined to be 1.46 ± 0.76 and 0.81 ± 0.44, respectively. To enhance contrast imaging, the mice were injected with two additional injections, each administrated 24 hr later. There was a noteworthy increase in tumor uptake, reaching 84.93 ± 2.68 ppm. The ratios of T/N and T/B were 25.20 ± 3.41 and 7.46 ± 0.66, respectively, for three consecutive injections (Figure 8B˗C). The obtained values were indicative of a tumor˗selective treatment which was deemed sufficient for therapeutic purposes. This improvement can be attributed to the enhanced permeability (EPR) and retention effects. The accumulation of boron and high T/B ratios indicated the promising efficiency of DSPE-BCOP-5T. The mice treated with DSPE-BCOP-5T and subsequent neutron irradiation demonstrated suppressed tumor growth through BNCT, highlighting the enhanced treatment effects achieved by DSPE-BCOP-5T 86.

Antibody˗based BNCT remains a promising treatment option. However, the prolonged blood circulation of antibodies has hindered their application. To address this issue copper free click chemistry was tested. In a study by Llop *et al.,* a pre˗targeting strategy for BNCT was attempted using trastuzumab. In this study, the monoclonal antibody (mAb) trastuzumab was modified with trans˗cyclooctene (TCO) to facilitate an *in vivo* click reaction with tetrazine˗installed; boron˗enriched gold nanoparticles (Tz˗B˗AuNPs). To facilitate the *in vivo* tracking, the AuNPs were radiolabeled with ^64^Cu 87. In subsequent experiments, mice with human breast cancer cells (BT-474) were divided into three groups: Group 1 received trastuzumab-TCO (100µg/mouse), Group 2 received trastuzumab without TCO conjugation, 24 h before the Tz˗B˗AuNPs injection; and Group 3 received only the Tz-AuNPs injection. The PET-CT scans quantification data for Group 3 exhibited high accumulation in the liver and spleen and a long residence time in the blood. The highest tumor uptake, reaching 4.76 ± 1.85 %ID cm^-3^, was observed at 24 h postinjection, indicating the passive accumulation of the tracer within the tumor due to EPR effect. Interestingly, both Groups 1 and 2 showed very similar biodistribution profile (4.42 ± 2.08 and 3.95 ± 0.38%ID cm^-3^, respectively). These findings indicate that the pre˗targeting strategy had a minimal impact on tumor targeting and persistence of radiolabeled tracers within the tumors. It was anticipated that rapid internalization of the mAb would limit the availability of mAb-TCO on the cell periphery, for the click reaction with Tz-AuNPs 87.

**Figure 9:**
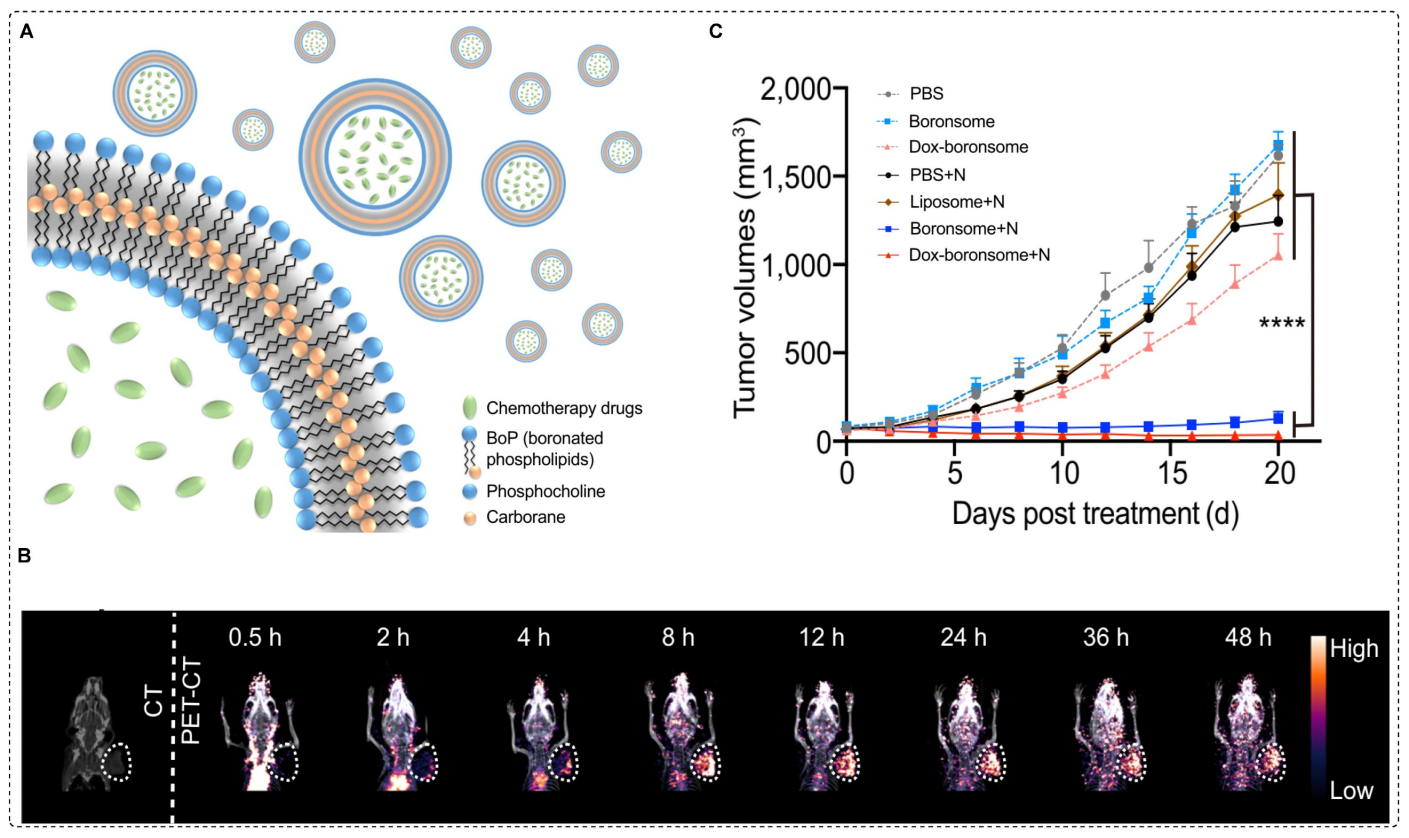
^ 64^Cu-NOTA-boronsome synthesis, uptake and preclinical evaluation. (A) Schematic illustration of boronated liposome loaded with chemotherapeutic drug (B) CT and PET/CT images of 4T1 tumor bearing mice using ^64^Cu-NOTA-boronsome, (C) Average tumor volume (mm^3^) (n = 9) of each group of mice utilizing various treatment process calculated up to 20 days post treatment, ****p < 0.001. Adapted with permission from 89, copyright 2022, Springer Nature Limited.

Clinically accepted small BNCT tracers have shown several limitations such as poor stability, low solubility, limited biocompatibility, and restricted boron delivery to tumors 88. To overcome these challenges, boron-containing nanocarriers have been investigated. Recently, boron˗enriched liposomes have been investigated for use in image˗guided BNCT and chemotherapy. In this approach, biocompatible and stable boronated liposomes were developed by covalently conjugating a carboranyl group to the hydrophobic region of phospholipids, resulting in a variety of boronated phospholipids (BoPs) through a thiol˗halo reaction. To form liposomal membranes, known as boronsomes, a boron-containing component was employed, specifically for the hydrophilic head. This arrangement resulted in an internal cavity with the capacity to accommodate additional drugs for synergistic therapy (Figure 9A). The boronsome exhibited long-term stability in 50% bovine serum at 37 °C. At a dosage of 5 mg/mL, the boron content in 4T1 cells reached a level as high as 182.5 ppm, effectively meeting the clinical requirements for BNCT. Remarkably, the boronsomes demonstrated superior cellular uptake compared to that of BPA, well-established boron delivery agent in clinical use, and FBY, a previously documented boronated amino acid derivative. Moreover, at concentrations of 5mg/mL or lower, the boronsomes exhibited excellent biocompatibility and efficacy, with more than 90% cell survival. Subsequently, [^64^Cu] Cu-NOTA-boronsomes were intravenously administered to mice bearing 4T1 tumors, and PET imaging was used to measure the boron concentrations in the organs of interest (Figure 9B). Boronsomes exhibited a tumor boron concentration of 93.3 ppm at 12 hr postinjection. The T/B ratio favored a neutron irradiation time of 12 hr. Biodistribution studies showed a tumor uptake of 5.49 ± 0.22 %ID/g with low uptake in surrounding tissues, indicating tumor selectivity. In comparison studies, boronsome outperformed BPA in terms of tumor boron enrichment and T/B ratio. Interestingly, mice treated with DOX loaded boronsome and subjected to thermal neutron irradiation exhibited remarkable suppression of tumor growth (Figure 9C) 89. The utilization of DOX loaded boronsome in BNCT treatment shows promising potential for future clinical studies.

### 4.3 ^89^Zr labeled BNCT tracers

BNCT has shown promising results in both preclinical and clinical investigations as potential treatment for head, neck, and brain tumors. Nevertheless, the utilization of BNCT for prostate cancer treatment remains elusive due to the absence of a suitable targeting probe 90. Recently, Flavell and co˗workers attempted a PSMA-based BNCT using PLGA-b-PEG conjugated ACUPA- and DFB- amphiphilic block co-polymer-based nanoparticles for a theranostic study of prostate cancer. ACUPA ligands targeting PSMA and polymers were also loaded with o-carborane to facilitate the delivery of boron to target cells for BNCT treatment 91. In Addition, the^89^Zr DFB ligands were incorporated for the PET imaging. ^89^Zr was chosen because its long half-life of 78.41 hr matches with the slow pharmacokinetics of the nanoparticles 92.

*In vitro* assays revealed that DFB (25)ACUPA(75) NPs exhibited remarkable specificity towards PSMA, with an IC50 value of 0.11 μg/mL, compared to 0.59 μg/mL for DFB (25)ACUPA(25) NPs. The high concentration of ACPUA ligands in the DFB (25) ACUPA (75) nanoparticles accounted for this phenomenon. Various *in vivo* assays demonstrated a relatively low tumor uptake of approximately 1%ID/g in PC3-Pip tumors. Interestingly, it was observed that the tumor uptake in PC3-Pip tumors was two˗folds higher than that of PC3-Flu tumors, which exhibited a value of approximately 0.5%ID/g. The radiotracer did not demonstrate a high affinity for *in vivo* targets despite the presence of ACUPA ligands. The disparity in tumor uptake can be ascribed to a multitude of factors, including the presence of distinct tumor-associated macrophages, heterogeneous pore sizes in tumors, and NP size. Furthermore, the observed differences in tumors may be partly influenced by passive accumulation facilitated by the EPR effects.

The radiolabeled NPs exhibited significantly higher uptake in the liver (100%ID/g), spleen (200 %ID/g), and kidneys (4 %ID/g). The significant bone uptake indicated nonspecific and weak chelation of ^89^Zr to nanoparticles with functional groups other than DFB chelators. These studies indicated that o-carborane molecules were detached from the PLGA core within 2 hr of injection and subsequently excreted from the bloodstream. Consequently, the tumor exhibits a remarkably low boron uptake. The suboptimal loading and release dynamics of carborane can be attributed to its rigid molecular structure, which limits its interaction with the PLGA units owing to its smaller surface area. Additionally, the weak non-covalent hydrophobic interactions between the carborane and the PLGA unit may also contribute to the suboptimal loading and release behavior. Overall, based on detailed biodistribution studies and PET/CT imaging, it was concluded that the tracer possessed inferior quality and was not suitable for use as a BNCT tracer to treat prostate cancer 91. In a previous study by Lolp and co˗workers, a pre˗targeting strategy was attempted using boron enriched ^64^Cu labeled AuNPs and trastuzumab antibodies. However, the desired therapeutic results have not been obtained. In another study, boron˗enriched carbon dots were selected as a pre˗targeting strategy, utilizing ^89^Zr labeled trastuzumab antibody. The carbon dots were chosen for their low toxicity, high biocompatibility, high boron loading capacity, and, more importantly, their ultra-small size, which facilitates rapid clearance from healthy tissues 93. Small boron˗rich carbon dots (B-CDs) were synthesized and decorated with tetrazine to produce B-CD-Tz for pre˗targeting studies. Similarly, the mAb was conjugated with TCO to produce trastuzumab-TCO. In some *in vivo* experiments,^89^Zr labeled trastuzumab-TCO was administrated to breast cancer xenograft models. PET images demonstrated that 24 hr postinjection was the optimal time point with the highest mAb accumulation in the tumor cells. Additionally,^18^F-labeled B-CD-Tz was used to study the biodistribution of and tumor targeting of B-CD-Tz. In the absence of trastuzumab-TCO, the tumor uptake was negligible. Furthermore, within 45 min, B-CD-Tz was cleared from the organs and a strong signal was detected in the bladder. Interestingly, when ^18^F˗labeled B˗CD˗Tz was injected 24 hr after trastuzumab-TCO, it exhibited significant tumor accumulation. The high tumor accumulation, which was observed 45 min after the administration of ^18^F˗labeled B˗CD˗Tz, confirmed the successful click reaction within the tumor. Although BNCT has not been studied in this particular case, it was concluded that the pre˗targeting strategy holds promise for future applications 93.

**Figure 10:**
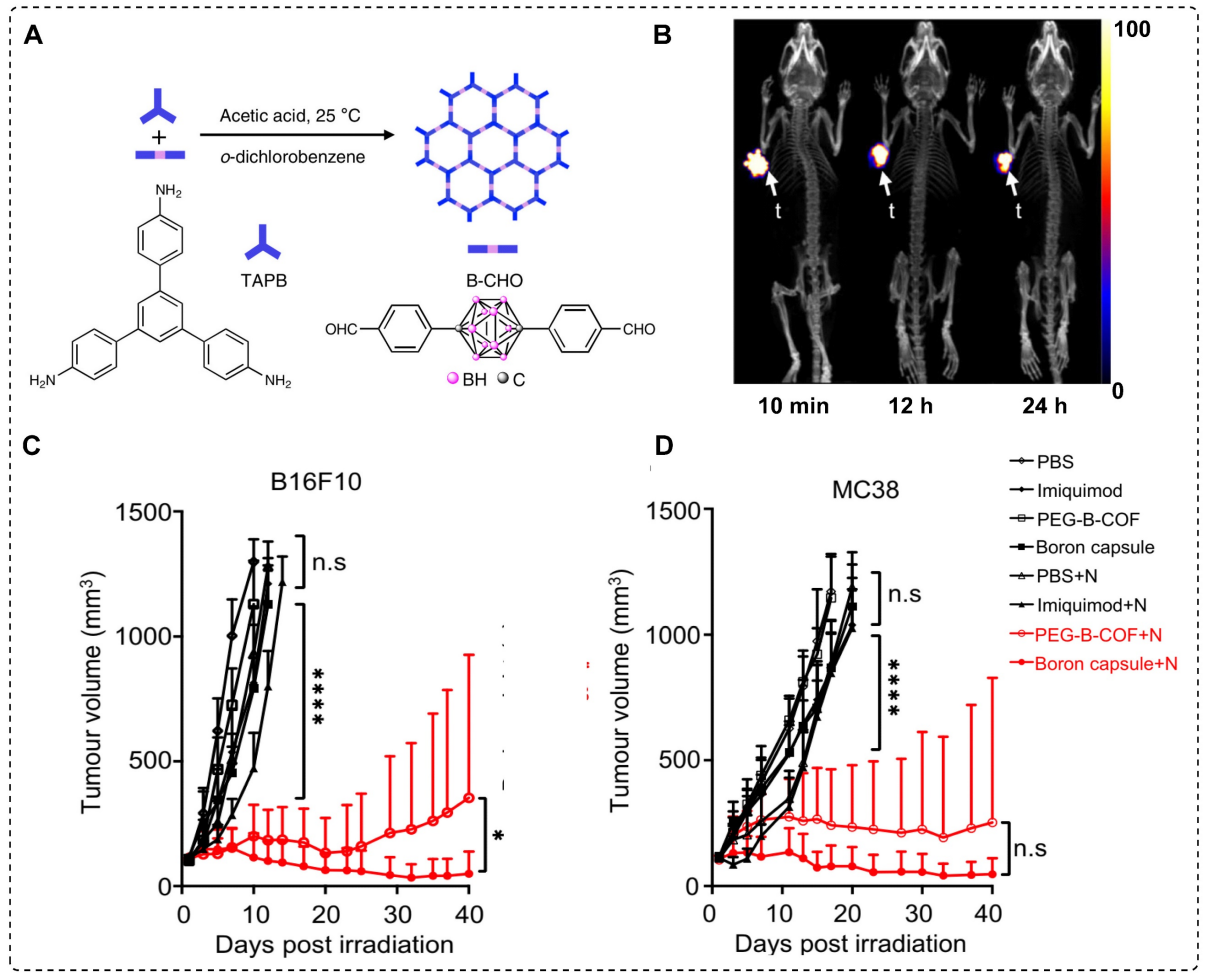
Schematic illustration of immune drug-loaded B-COF particles and preclinical studies. (A) Schematic representation of the synthesis of carborane-derived covalent organic framework (B˗COF) by the condensation of 1, 3, 5-tris (4-aminophenyl)-benzene (TAPB) and p-carborane-1, 10-phenyl-dialdehyde (B˗CHO) under an optimized condition. (B) PET/CT images of B16F10 tumor-bearing mice at an indicated time point after intratumoral injection of ^89^Zr˗Boroncapsule (n=3). (C, D) Average tumor volume (mm^3^) (n = 6) of each group of mice utilizing various treatment processes calculated up to 40 days post treatment, ****p < 0.0001 with, (C) B16F10, and (D) MC38 xenograft. Adapted with permission from 97, copyright 2022, Springer Nature Limited.

Combined chemotherapy and BNCT are likely to offer numerous advantages, particularly if superior tracers with excellent drug release capabilities are used 94. Controlled drug release has gained attention in the field of covalent organic frameworks (COF) 95. This exceptional interest can be attributed to several characteristics of COF, including periodic skeletal structures, substantial specific surface area, biological stability, and uniform pore sizes, which guarantee efficient loading and controlled release of the drug 96. Based on this idea, a carborane-based covalent organic framework (B-COF) was explored for concurrent immunotherapy and BNCT. To achieve high and sustained levels of boron in the tumor for an extended period, as well as controlled delivery of an immune drug, boron capsules in the form of µm-scale B-COF were synthesized. To enhance the *in vivo* stability and achieve uniform distribution, immune drug-loaded B-COF particles were coated with DSPE-PEG, resulting in the formation of PEG-B-COF particles (Figure 10A). Significant growth inhibition was observed in both MC38 and B16F10 cancer in previous *in vitro* studies using boron capsules and PEG-B-COF in combination with thermal neutron treatment. For comparison, boron capsules were radiolabeled with ^89^Zr to obtain^89^Zr-boron capsules, which were injected intratumorally into the mice. The vast majority of ^89^Zr-boron capsules exhibited remarkable tumor retention within 24 hr, with negligible leakage into the circulatory system or surrounding tissues (Figure 10B). Subsequently, mice bearing B16F10 cancer cell xenograft were treated with boron capsules on day 6, followed by sequential neutron irradiation on day 7. Mice treated with boron capsules or neutron irradiation alone displayed mixed patterns of tumor growth. However, compared with the control group, the combination of boron capsules and neutron irradiation led to a significant 40-day growth delay. Remarkable inhibition of tumor growth was observed in mice treated with PEG-B-COF; however, the efficacy of the treatment varied among individual mice, indicating heterogeneity in the response (Figure 10C-D). This observation suggests that the release of an immune adjuvant induced by neutron irradiation may be crucial for achieving a comprehensive tumor treatment. Additionally, mice engrafted with the MC38 mouse tumor cell line exhibited a similar tumor regression effect when treated with the combination of boron capsules and neutron irradiation 97.

### 4.4 ^67/68^Ga labeled BNCT tracers

**Figure 11:**
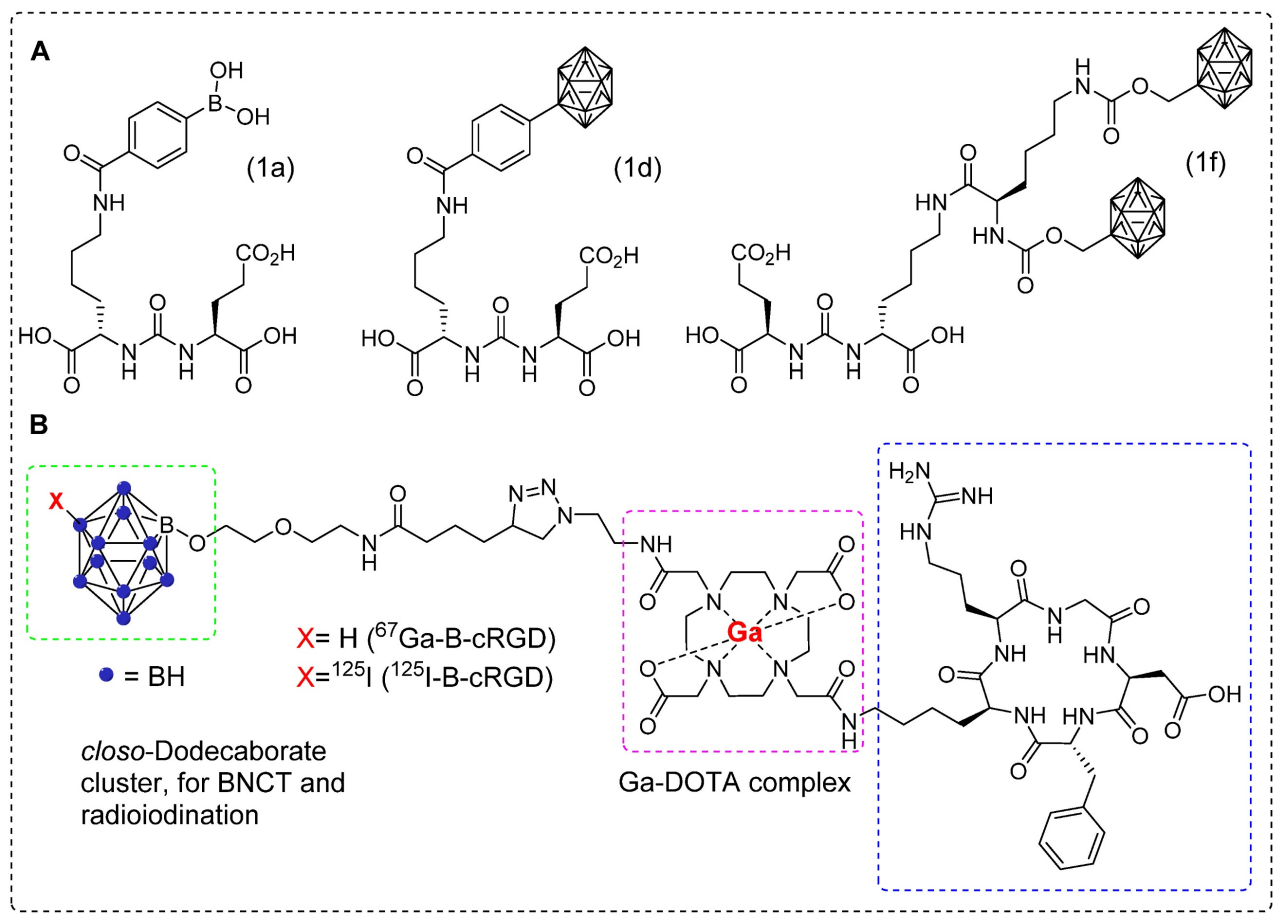
Representative boron-installed radiolabeled PSMA and cyclic RGD agents. (A) Schematic illustration of selected boron installed PSMA agents 1a, 1d, and 1f. (B) Schematic illustration of boron-installed cyclic RGD peptide^67^Ga-B-cRGD and ^125^I-B-cRGD. Adapted with permission from 102, copyright 2022, American Chemical Society.

^68^Ga is produced using a generator system, which enables the efficient production of radiopharmaceuticals for PET imaging. PET imaging with ^68^Ga offers high˗resolution images, allowing for the precise localization and characterization of abnormalities, thereby aiding in the disease detection and assessment of treatment response 98, 99. ^68^Ga-PSMA is recognized as one of the most effective radiopharmaceuticals for prostate cancer 100. Recently, a series of boron-installed PSMA˗based BNCT tracers, incorporating either carborane or boronic acid functional groups were synthesized. The three tracers were selected for further evaluation after the initial screening. Compounds 1a (boronic acid) and 1d (carborane) were selected as representative compounds based on their characteristics. Compound 1f was selected for its two carborane substituents, highest boron atom count (20), and favorable binding affinity (Figure 11A). All three compounds demonstrated high binding affinity to serum proteins and exhibited negligible cell toxicity. For the *in vivo* experiments, mice bearing 22Rv1 tumor xenograft were used in both the ^68^Ga-PSMA-11 blocking assay and boron biodistribution studies. The results were compared with those of BPA. Micro PET/CT images revealed tumor uptake of 0.119 ± 0.071, 0.117 ± 0.007, 0.564 ± 0.098, and 1.896 ± 0.408 %ID/g for compounds 1a, 1d, 1f, and BPA, respectively. The T/N and T/B ratios decreased with the administration of the inhibitor. At 4 hr postinjection, the most favorable outcomes were observed. Notably, compound 1d demonstrated a remarkable tumor boron uptake of 4.2 μg/g of tissue, approximately twice as high as the boron uptake in muscles (2.2 μg/g of tissue). These findings highlight the superior performance of compound 1d after 4 hr, indicating its potential use as an effective treatment agent. In the case of compound 1f, both at 4 and 1 hr, the tumor uptake was measured to be 3.4 μg/g of tissue, which was lower than the corresponding boron uptake in muscle (5.1 μg/g of tissue for both time points). Compounds 1d and 1f exhibited similar levels of boron uptake in the tumor as those of BPA. *In vivo*, the hydrophilic nature of compound 1a facilitated its rapid elimination through renal clearance, while simultaneously exhibiting strong binding to PSMA. However, despite the highest administered dose, compound 1a exhibited lower tumor boron uptake at 4 hr, may be attributed to its molecular structure, which consists of only a single boron atom per molecule. Overall, the tumor boron uptake did not meet the required levels (20˗50 μg/g) for effective BNCT, and the T/N ratio was below the desired selectivity (less than 3:1) 101. In 2022, Ogawa and co˗workers developed closo˗dodecaborate installed cyclic RGD peptide (B-cRGD) for targeted and efficient tumor treatment through BNCT. To track the tracer *in vivo*, a DOTA chelator was incorporated for radiolabeling using ^67^Ga (t_1/2_ = 3 d), resulting in ^67^Ga-B-cRGD.Additionally, iodine-125 (^125^I) was introduced in to the closo˗dodecaborate moiety to synthesize ^125^I-B-cRGD for comparison purposes (Figure 11B). In the *in vitro* experiment, both tracers exhibited significant uptake by U-87 MG human glioblastoma cells for up to 6h. Biodistribution studies were performed using a mouse model of U-87MG xenograft. Both tracers displayed high tumor uptake and rapid clearance from the healthy tissues. However, ^125^I-B-cRGD demonstrated higher tumor uptake than that of ^67^Ga-B-cRGD because of its higher binding capacity to albumin. This study did not provide information regarding the quantitative analysis of boron levels in target tissues or the evaluation of the treatment efficacy resulting from the application of thermal neutron irradiation 102.

## 5. SPECT Imaging of BNCT tracers

### 5.1 ^123^I labeled BNCT tracers

**Figure 12:**
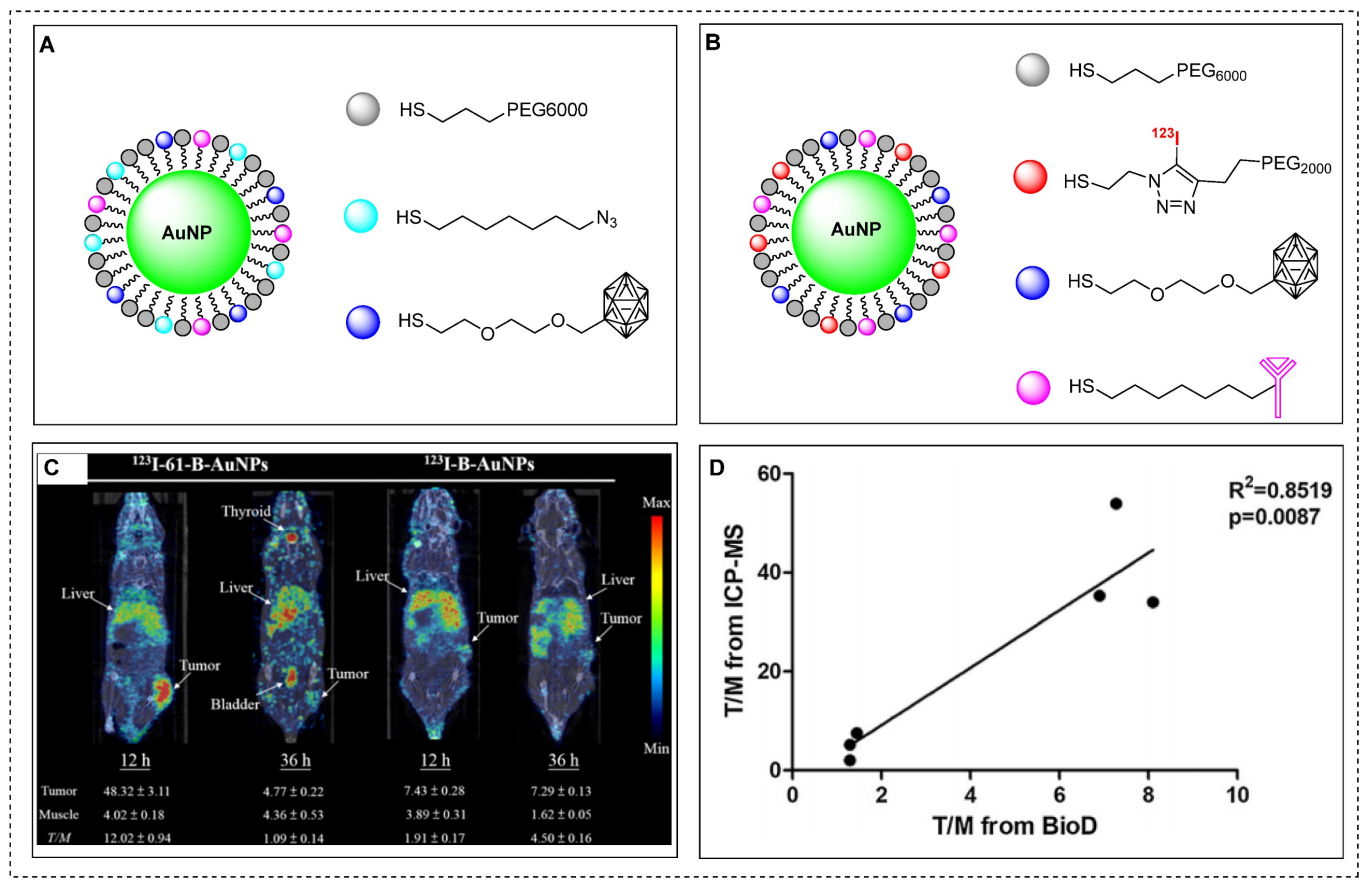
Representative images of ^123^I-61-B-AuNPs and preclinical studies. (A) Schematic illustration of gold nanoparticles functionalized with boron cages (B-AuNPs). (B) B-AuNPs conjugated to anti-HER2 antibody (61IgG) to form 61-B-AuNPs and radiolabeled with ^123^I to yield^123^I-61-B-AuNPs. (C) SPECT/CT images of N87 xenograft-bearing mice intravenously injected with^123^I-B-AuNPs or ^123^I-61-B-AuNPs at 12 and 36 hr postinjection. (D) Correlation between the tumor-to-muscle ration obtained from biodistribution studies using gamma counter and that derived from boron content determined by ICPMS (R^2^ = 0.8519, p < 0.05). Adapted with permission from 105, copyright 2019, Elsevier B.V.

Gold nanoparticles (AuNPs) are considered as excellent platforms for selective delivery of anticancer drugs to tumors 103. Previously, boron˗enriched AuNPs were synthesized and tested for successful BNCT treatment of tumors. However, there are still limitations in understanding the *in vivo* biodistribution, pharmacokinetics and T/B ratio using non˗invasive imaging techniques 104**.** Recently, theranostic assemblies of AuNPs functionalized with boron cages (B-AuNPs) were developed and conjugated to an anti-HER2 antibody (61 IgG) to form 61-B-AuNPs. To investigate the cellular uptake in vitro and assess the pharmacokinetics *in vivo* using SPECT-CT imaging, radioactive iodine (^123^I) was incorporated in to B-AuNPs or 61-B-AuNPs using copper-catalyzed click chemistry (Figure 12A-B) 105. Over time, ^123^I-61-B-AuNPs gradually increased the cellular uptake to a maximum level of 23.31 ± 4.84, 36 hr post-incubation. In contrast, ^123^I-B-AuNPs showed limited uptake (0.97 ± 0.05) due to the absence of HER2-targeting antibodies. ^123^I-61-B-AuNPs demonstrated improved cellular internalization over time, increasing from 10.53 ± 1.21% at 12 h to 15.40 ± 1.72% at 36 hr post-incubation, while ^123^I-B-AuNPs was not internalized into the cells. SPECT/CT imaging of mice that were administrated with ^123^I-61-B-AuNPs or^123^I-B-AuNPs revealed a more noticeable accumulation of the former compound. The tumor uptakes for^123^I-61-B-AuNPs and^123^I-B-AuNPs were 48.32 ± 3.11 and 7.43 ± 0.28%ID/mL, respectively, at 12 h postinjection (Figure 12C). The mice injected with ^123^I-61-B-AuNPs demonstrated a higher T/M ratio of 7.42 ± 0.61, in comparison to mice injected with ^123^I-B-AuNPs (T/M ratio 1.34 ± 0.09). The intratumoral boron concentrations in mice injected with ^123^I-61-B-AuNP and ^123^I-B-AuNP were 217.1 ± 47.1 and 74.5 ± 6.0 μg/g, respectively. The pharmacokinetics and tumor accumulation of ^123^I-61-B-AuNP, as demonstrated by SPECT-CT images were effectively correlated with the boron content in specific organs and tumors (Figure 12D). This suggests that imaging tracer based on AuNPs can be used to predict the treatment efficiency using BNCT.

Overall, PET and SPECT imaging technologies offer valuable solutions to address the challenges associated with BNCT. One significant concern in BNCT is the heterogeneous distribution of boron within and around the tumor, which leads to inaccuracies in the dose distribution.Moreover, incomplete excretion of the administrated dose from healthy tissues or excessive dose deposition in the tumor can potentially trigger secondary cancer 106. To overcome these inherent limitations of BNCT, PET and SPECT provide promising alternatives by enabling precise quantification of boron uptake in tumors and neighboring healthy organs.

Recently, radiolabeling of BNCT tracers and the application of molecular imaging to study the *in vivo* chemical and pharmacological behavior have further enhanced this treatment procedure. However, it is important to note that most of the studies summarized in this review are based on preclinical data; thus, more comprehensive clinical studies are required to understand the potential of molecular imaging in combination with BNCT. At present, ^18^F-FBPA is being employed in clinical settings to assess boron distribution in patients 107-109, with the expectation that forthcoming radiolabeled BNCT tracers will be integrated into clinical research endeavors, further catalyzing the advancement of this therapeutic strategy.

## 6. Conclusion and future perspectives

The global cancer burden emphasizes the need for optimal, effective, and safe treatment modalities. BNCT has emerged as a promising approach; however, clinical practice primarily relies on the use of BPA, BSH, and their analogs, as boron˗delivery tracers. This has encouraged scientific interest in the exploration of new and improved tracers. The efficiency of BNCT depends upon several critical factors, including the pharmacokinetic behavior of the boron˗enriched tracer, its affinity for the tumor tissue, the spatial biodistribution of boron and the optimal timing for neutron irradiation. Innovative imaging techniques, such as PET or SPECT can be employed to ensure precise and noninvasive determination of boron content in tumors. Several radioisotopes, such as ^18^F, ^64^Cu, ^89^Zr, ^68^Ga, and ^123^I, have been used for radiolabeling and molecular imaging of BNCT tracers. This progress has enabled a comprehensive understanding of the *in vivo* behavior and targeting ability of the new tracers.

Radiolabeling of BNCT tracers shows tremendous potential for accurately quantifying boron in tumors. However, there is an urgent need for developing novel radiolabeled BNCT tracers to improve pharmacokinetics, enhance treatment precision, and reduce potential risks of secondary cancer. Additionally, exploring innovative targeted drug delivery systems can increase the concentration of boron in the tumor tissues while sparing the healthy organs; thus, maximizing the treatment effectiveness. In-depth radiobiological studies are required to better understand the interaction between boron and thermal neutron radiation, as well as the mechanism governing secondary cancer formation. Furthermore, conducting comprehensive evaluation of the cost-effectiveness of PET and SPECT imaging in BNCT compared to other treatment modalities hold significant promise in guiding healthcare policies and resource allocation decisions. Designing robust guidelines and criteria for patient selection and incorporating molecular imaging assessments are critical aspects that can significantly improve treatment outcomes and resource utilization. Looking ahead, extensive research on long-term patient monitoring using PET or SPECT imaging coupled with comprehensive clinical studies to validate the efficiency and safety of new BNCT tracers are vital for advancing the field and implementing this cutting-edge technology in cancer care. By focusing research efforts on these well-structured paths, the integration of PET and SPECT imaging into BNCT holds the potential to revolutionize personalized and effective cancer treatments.

## Figures and Tables

**Table 1:** Important tracers for targeted ^10^B delivery in boron neutron capture therapy.

Tracer	T/B^*^ ratio	^10^B in tumor (μg/g)	Tumor type	Ref.
Boronophenylalanine (BPA)	2.5-3.0	34.0-99.0	Glioma	10
Sodium mercaptoundecahydro-closo-dodecaborate (BSH)	2.5-3.0	17.5-41.0	Glioma	11
Boronophenylalanine-fructose	2.5-3.0	15.0-34.0	Multiforme Glioblastoma	12
1-amino-3-borono-cyclopentanecarboxylic acid (cis-ABCPC)	16.4-16.5	16.4-16.5	Melanoma	13
3-[5-{2-(2,3-Dihydroxyprop-1-yl)-o-carboran-1-yl}pentan-1-yl]thymidine(N5-2OH)	-	16.2-16.5	Giloma (F98)	14
Cu(II) complexed porphyrins (CuTCPH)	>100	62.0-62.5	EMT-6/Cub or EMT-6/RO	15
Zn(II) complexed porphyrins (ZnTCPH)	>105	137.0-138.0	EMT-6/Cub or EMT-6/RO	15
Porphyrins (H2TBP)	175.25	61.9-78.3	Giloma (F98)	16
Tetrakis-carborane carboxylate ester of 2,4-bis-(a,b-dihydroxye thyl)-deutero-porphyrin IX (BOPP)	1855.4	518.0-617.5	Brain tumor	17
Boronated Porphyrin Nanoparticles (BPN)	33.88	125.18±14.54	4 T1	18
Boronated starburst dendrimer (BSD)-EGF bioconjugates	>100	21.1-25.5	Giloma (F98)	19
Boronophenylalanine-Tyrosine (BPA-Tyr)	2.0-2.1	7.21-7.51	As PC-1	20
Cetuximab-boronated polyamidoamine dendrime (BD-C225)	>100	77.0-91.2	Giloma (F98)	21
L8A4 mAb-boronated polyamidoamine dendrime (BD- L8A4)	>100	32.0-35.5	Giloma (F98)	22
Transferrin-PEG liposomes	6.0-7.0	35.0-36.5	Colon 26	23
MAC and TAC-liposomes	1.8-20	67.0-67.8	EMT6	24
Poly(L-lactide-coglycolide) coated nanoparticles (PLLGA)	3.0-5.0	113.9-128.9	-	25
Poly(ethylene glycol)- b-poly(glutamic acid) [PEG-b-P(Glu)] conjugated with PEG-b-P(Glu-SS-BSH)	20	70.0-90.1	C 26	26
^10^B-enriched nanoparticles (^10^BSGRF NPs)	2.0-2.8	50.0-50.5	Brain	27
Single-walled carbon nanotubes (SWCNTs)	3.0-3.12	22.0-22.8	EMT 6	28
Boron nitride nanotubes (BNNTs)	n.d.	n.d.	GMB cells	29
Folic acid functionalized boron phosphate (BPO_4_) nanoparticles	n.d.	n.d.	Cytotoxicity study, colon and osteosarcoma cell line study	30

^*^Tumor -to-blood Boron-10 concentration ratio

**Table 2:** Selected radioisotopes used in molecular imaging and potential application in BNCT

Radionuclide	Half-life	Mode of decay	Imaging	Tested in BNCT	Future application
Fuorine-18	109 min	Beta (+) decay	PET	Tested at preclinical and clinical studies	Suitable for small BNCT tracers or indirect radiolabeling
Zirconium-89	78.4 hr	Beta (+) decay	PET	Tested at preclinical studies	Suitable for antibodies, protein, or nanomaterials based BNCT tracers
Copper-64	12.7 hr	Beta (+) decay	PET	Tested at preclinical studies	Suitable forprotein, peptide, based BNCT tracers
Gallium-68	68 min	Beta (+) decay	PET	Tested at preclinical studies	Suitable for small BNCT tracers or indirect radiolabeling
Iodine-124	4.2 days	Beta (+) decay	PET	Not yet	Suitable for antibodies, protein, or nanomaterials based BNCT tracers
Manganese-52	5.2 days	Beta (+) decay	PET	Not yet	Suitable for antibodies, protein, or nanomaterials based BNCT tracers
Iodine-123	13.3 hr	Electron capture	SPECT	Tested at preclinical studies	Suitable forprotein, peptide, or nanomaterials based BNCT tracers
Indium-111	2.8 days	Electron capture	SPECT	Not yet	Suitable for antibodies, protein, or nanomaterials based BNCT tracers
Rhenium-188	17 hr	High energy beta	SPECT	Not yet	Suitable forprotein, peptide, or nanomaterials based BNCT tracers
Iodine-125	59.4 days	Electron capture	SPECT	Not yet	Suitable for antibodies, protein, or nanomaterials based BNCT tracers
Technetium-99m	6.0 hr	Isomeric transition	SPECT	Not yet	Suitable for small BNCT tracers

**Table 3:** Selected radioisotopes and their uptake in healthy tissues or tumors as free elements

Isotope	Tumor	Liver	Bone	Kidneys	Blood	Thyroid	Stomach	Heart	Salivary Glands
^18^F			Δ						
^89^Zr			Δ						
^64^Cu	Δ	Δ		Δ					
^68^Ga	Δ	Δ	Δ	Δ	Δ				
^124^I						Δ	Δ		Δ
^52^Mn	Δ	Δ		Δ				Δ	Δ
^123^I						Δ	Δ		Δ
^111^In	Δ	Δ	Δ	Δ					
^188^Re						Δ	Δ		Δ
^125^I						Δ	Δ		Δ
^99m^Tc						Δ	Δ		Δ

## Abbreviations

AuNPs: gold nanoparticles
B-CDs: boron-rich carbon dots
B-COF: carborane-based covalent organic framework
BNCT: boron neutron capture therapy
BoPs: boronated phospholipids
BPA: (L)-4-dihydroxy-borophenylalanine
BPNs: boronated porphyrin nanoparticles
BSH: sodium mercaptoundecahydro-closo-dodecaborate
COF: covalent organic frameworks
COPs: covalent organic polymers
DSPE-PEG: 1,2-distearoyl-sn-glycero-3-phosphoethanolamine-N-[amino-(polyethyleneglycol)-2000]
EPR: enhanced permeability and retention
FBY: fluoroboronotyrosine
FDG: fluorodeoxyglucose
L-BPA: structural analog of boronophenylalanine
LET: linear energy transfer
mAb: monoclonal antibody
PET: positron emission tomography
SPECT: single-photon emission computed tomography
T/B: tumor-to-blood
T/N: tumor-to-normal
TCO: trans-cyclooctene
Tz-AuNPs: tetrazine-installed boron-enriched gold nanoparticles
